# Development of a diagnostic algorithm identifying cases of dislocation after primary total hip arthroplasty—based on 31,762 patients from the Danish Hip Arthroplasty Register

**DOI:** 10.1080/17453674.2020.1868708

**Published:** 2021-01-13

**Authors:** Lars L Hermansen, Bjarke Viberg, Søren Overgaard

**Affiliations:** a Department of Orthopedics, Hospital of South West Jutland, Esbjerg;; b The Orthopedic Research Unit, Department of Orthopedic Surgery and Traumatology, Odense University Hospital, Odense, Department of Clinical Research, University of Southern Denmark;; c OPEN, Odense Patient data Explorative Network, Odense University Hospital, Odense;; d Department of Orthopedic Surgery and Traumatology, Lillebaelt Hospital, University Hospital of Southern Denmark;; e Department of Regional Health Research, University of Southern Denmark, Denmark

## Abstract

Background and purpose — Dislocation of total hip arthroplasties (THA) is often treated with closed reduction and traditionally not registered in orthopedic registers. This study aimed to create an algorithm designed to identify cases of dislocations of THAs with high sensitivity, specificity, and positive predictive value (PPV) based on codes from the Danish National Patient Register (DNPR).

Patients and methods — All patients (n = 31,762) with primary osteoarthritis undergoing THA from January 1, 2010 to December 31, 2014 were included from the Danish Hip Arthroplasty Register (DHR). We extracted available data for every hospital contact in the DNPR during a 2-year follow-up period, then conducted a comprehensive nationwide review of 5,096 patient files to register all dislocations and applied codes.

Results — We identified 1,890 hip dislocations among 1,094 of the included 31,762 THAs. More than 70 different diagnoses and 55 procedural codes were coupled to the hospital contacts with dislocation. A combination of the correct codes produced a sensitivity of 63% and a PPV of 98%. Adding alternative and often applied codes increased the sensitivity to 91%, while the PPV was maintained at 93%. Additional steps increased sensitivity to 95% but at the expense of an unacceptable decrease in the PPV to 82%. Specificity was, in all steps, greater than 99%.

Interpretation — The developed algorithm achieved high and acceptable values for sensitivity, specificity, and predictive values. We found that surgeons in most cases coded correctly. However, the codes were not always transferred to the discharge summary. In perspective, this kind of algorithm may be used in Danish quality registers.

Dislocation is a feared complication after total hip replacement. To prevent this and other complications, we often have to take advantage of and rely on the enormous amount of data from the many orthopedic registries in use rather than conducting large and costly clinical studies (SHAR 2017, AOANJRR 2018, DHR 2019, Varnum et al. 2019a, b).

However, data within these registries are not always representative of the actual occurrence of complications. Gundtoft et al. (2015) demonstrated that infections are underreported by 40%. Likewise, a recent study based on a Danish cohort and administrative registers found that the sensitivity was only 63% when patients with dislocations were identified through a combination of the correct diagnosis and procedure code, ultimately missing more than one-third of the patients (Hermansen et al. 2020). The treatment of choice after reduction of the first hip dislocation is nonoperative, unless there is obvious malpositioning of the inserted components causing instability. Revisions are often performed only after several dislocations (Patel et al. 2007, Devane et al. 2012, Saiz et al. 2019). Therefore, a large group of patients treated with closed reduction are never registered in arthroplasty registers and the true burden of this complication remains uncertain.

Ideally, a healthcare system should be able to capture all important complications that have an impact on the patient and the treatment quality. This study aimed to create an algorithm to identify dislocations of THAs with high sensitivity, specificity, and predictive values based on codes from a national health care system.

## Patients and methods

### Study design

We used data that was collected during a recent retrospective cohort study which used prospectively collected data from the Danish Hip Arthroplasty Register (DHR) designed to find the true frequency of hip dislocation after primary THA (Hermansen et al. 2020). We refer the reader to this paper for study details and will only report the main aspects in this article. The RECORD guidelines were followed.

### Participants (Figure 1)

We identified all patients with primary osteoarthritis (OA) who underwent a THA from January 1, 2010 to December 31, 2014 and followed each patient for 2 years after index surgery. Follow-up was ended after 2 years or before if revision surgery, emigration, or death occurred, whichever came first. We excluded THAs inserted for indications other than primary OA. For the same reason, patients younger than 40 years of age were excluded (Duffy et al. 2001, Ellison et al. 2006). Any contralateral THA procedures during the inclusion period was also omitted to avoid dependency among observations (Ranstam and Robertson 2010).

### Data sources and data cleaning

Dislocations, together with any other type of patient contact with the Danish healthcare system, are registered in the Danish National Patient Register (DNPR) (Lynge et al. 2011). By means of the DNPR, we were able to extract information for every hospital contact with orthopedic and non-orthopedic departments as well as outpatient emergency room contacts for each patient during the individual 2-year follow-up period. We extracted the admission and discharge date, the date of any surgical procedure, and hospital and department names for all hospital contacts that had been assigned any primary or secondary hip or dislocation related diagnostic or procedural code (see Appendix for the complete list). The DNPR completeness is over 99%, and we did not encounter any missing data regarding diagnoses and procedure codes in our population (Schmidt et al. 2015).

The diagnostic codes were extracted from the International Classification of Diseases, 10th revision (ICD-10) and procedural codes were derived from the Danish version of the Nordic Medico-Statistical Committee’s (NOMESCO) Classification of Surgical Procedures (NCSP). We then established the following classification of contacts:

1. Genuine dislocations: Contacts assigned a combination of the correct diagnostic (DT84.0(A)) and surgical procedure (KNFH20) codes.
2. Possible dislocations: Any contact not included as a genuine dislocation.

A comprehensive review of all patient files meeting criterion 2 was performed to identify every miscoded dislocation. We also reviewed 20% of the genuine dislocation cases to validate the combination of correct codes. 5,096 patient files were manually reviewed, and all dislocations and the applied codes were registered.

### Statistics

We designed the algorithm using a stepwise approach and calculated the sensitivity, specificity, and the positive and negative predictive values for various combinations of the most frequently used codes. The steps were not pre-specified but, instead, were chosen based on the codes that had been applied nationwide from 2010 to 2016 for verified dislocations. The plan was to add codes in steps and continuously increase the sensitivity (i.e., the proportion of true positives of all dislocations), while at the same time keeping the specificity (i.e., the proportion of true negatives of all not having a dislocation) and the positive predictive value (PPV) (i.e., probability that patients based on the algorithm truly have the dislocation) high. The algorithm will identify patients with at least 1 episode of dislocation for a given period of time (i.e., the risk of dislocation) but it will not necessarily identify all dislocations for each patient. It is also important to note that there is a clear distinction between hospital contacts with or without denoted laterality in the DNPR. This is an important aspect in order to distinguish between contralateral THAs. Statistics was performed with STATA software version 15.0 (StataCorp, College Station, TX, USA).

### Ethics, funding, and potential conflicts of interest

The Danish Patient Safety Authority (3-3013-2128/1), the Danish Data Protection Agency (2008-58-0035), and the head of local departments approved the review of patient medical records. Funding was obtained from the Clara Hansens Memorial Fund, Appropriation Merchant Sven Hansen & Wife Ina Hansens Foundation, the Danish Rheumatism Association, the A.P. Møller Foundation for the Advancement of Medical Science, the Orthopaedic Fund of West Jutland, and Doctor of Bramming, Grethe Marie Justesens Fund. The University of Southern Denmark and Region of Southern Denmark each assigned a 1-year PhD scholarship. There are no conflicts of interest and none of the funding had any influence on the data material or reporting of the results.

## Results

**Figure 1.**
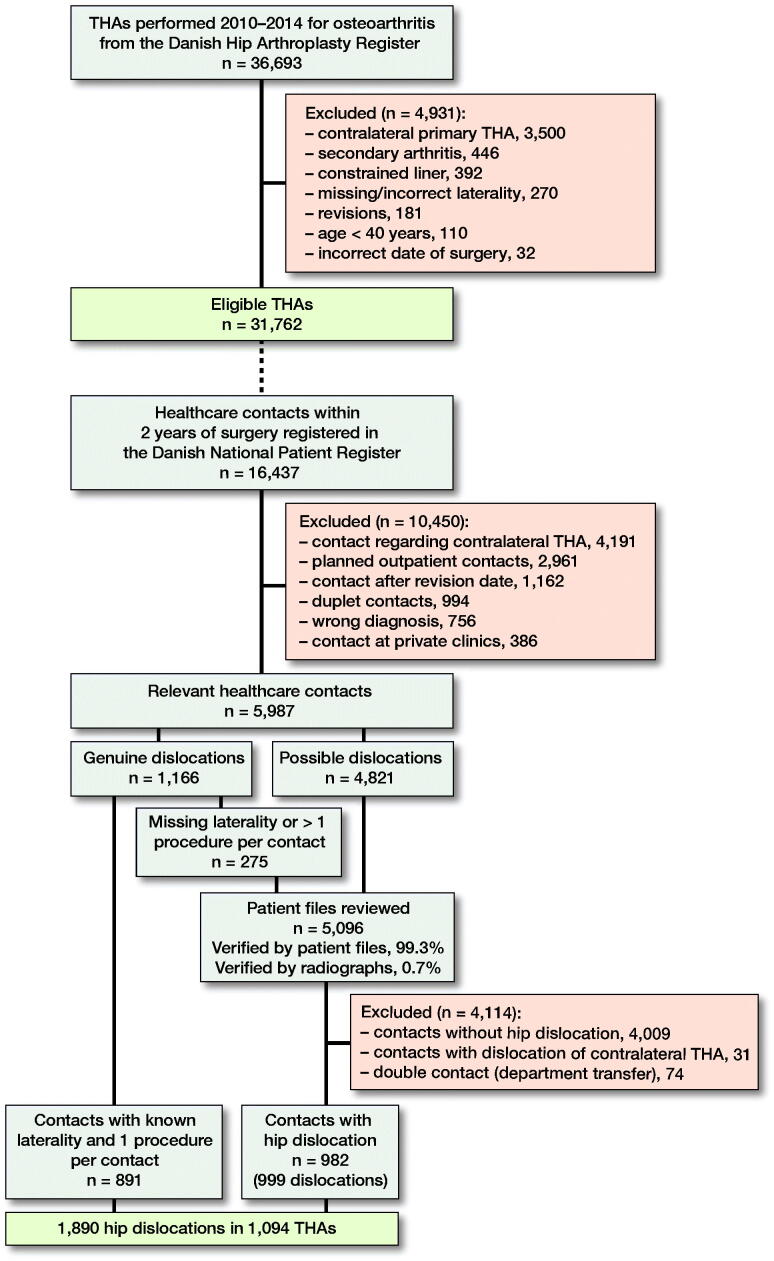
Flowchart overview of DHR (upper part) and DNPR (lower part) data cleaning and the following patient file review to identify the true frequency of dislocations. The dotted line indicates that 16,437 hospital contacts were found in the DNPR for the 31,762 included THAs.

We identified 1,890 hip dislocations in 1,094 of the included 31,762 THAs (Figure 1), which yielded a 2-year cumulative incidence of 3.4% (95% CI = 3.3–3.6). More than 70 different main diagnoses and 55 different procedural codes were coupled to the hospital contacts with dislocation. The most common mistake was the application of the correct procedure code in combination with the wrong diagnosis code. Thereafter, more than 10% of all dislocations were found to have codes that are intended to describe dislocation and reduction of traumatic hip dislocation of native hip joints rather than THA.

The most frequently used codes and combinations were grouped and contributed to our algorithm (Table 1). Step 1 was a combination of the correct codes (DT840 + KNFH20) with known laterality resulting in a sensitivity of 63% and a PPV of 98% (Table 2). When we added the contacts with the correct procedure code alone (KFH20) and alternative and often applied codes in 2 additional steps (DS730, KNFH (21;22;00;02)), all with known laterality, we increased the sensitivity to 85%, while the PPV was 96%. Step 4 added the contacts of the above-mentioned codes from steps 1 through 3 with unknown laterality in the DNPR, which increased the sensitivity to 91% but lowered the PPV to 93%.

**Table 1.** Description of the 5 groups of diagnostic combinations used in the algorithm

Group	Codes	Description
Group 1	DT840(A) + KNFH20	Combination of correct diagnosis and procedure code (with identified laterality in DNPR)
Group 2	KNFH20	Correct procedure code alone combined with any random diagnosis (with identified laterality in DNPR)
Group 3	DS730	Alternative and often used diagnosis and procedure codes (with identified laterality in DNPR)
	KNFH00	
	KNFH02	
	KNFH21	
	KNFH22	
Group 4	Group 1–3	All group 1–3 cases, where laterality is uncertain in DNPR
Group 5	DT840(A)	Correct diagnoses alone combined with any random procedure code, AND limited to acute readmissions or emergency room
		contacts (with identified laterality in DNPR)

See Appendix for detailed definitions of diagnostic and procedure codes.

**Table 2.** Sensitivity, specificity, positive predictive value (PPV), and negative predictive value (NPV) for each step of the algorithm identifying dislocations. Values are % with (95% confidence interval)

Step	Sensitivity	Specificity	PPV	NPV
Step 1	62.7 (59.8–65.6)	99.9 (99.9–99.9)	97.9 (96.5–98.8)	98.7 (98.6–98.8)
Step 2	77.0 (74.4–79.4)	99.9 (99.9–99.9)	96.5 (95.0–97.6)	99.2 (99.1–99.3)
Step 3	85.1 (82.9–87.2)	99.9 (99.8–99.9)	96.3 (94.9–97.4)	99.5 (99.4–99.5)
Step 4	91.3 (89.5–92.9)	99.8 (99.7–99.8)	93.3 (91.6–94.7)	99.7 (99.6–99.7)
Step 4A	91.3 (89.5–92.9)	99.9 (99.8–99.9)	96.5 (95.2–97.6)	99.7 (99.6–99.7)
Step 5	95.4 (93.9–96.5)	99.2 (99.1–99.3)	81.8 (79.4–83.7)	99.8 (99.8–99.9)
Step 5A	95.4 (93.9–96.5)	99.8 (99.8–99.9)	96.6 (95.4–97.7) ^a^	99.8 (99.8–99.9)

Step 1 = Group 1 alone; Step 2 = Group 1 + 2 etc. For each step an additional group is added to the previous step, thereby including more codes and increasing sensitivity at a cost of decreased specificity and the positive predictive value. The two steps marked (A) indicate how Steps 4 and 5 can achieve an increase in the positive predictive value if the patient files for the hospital contact of the specific group are reviewed and the false-positives are discarded. See appendix for examples of the review burden.

**^a^**Assumes patient file review of Step 4.

The only way to increase the sensitivity further was to include contacts with the correct diagnosis code alone (DT840). However, this code is often related to many other aspects of prosthesis complications and is not used solely for dislocations. Therefore, in the last step, the sensitivity increased up to 95% but at the expense of an unacceptable decrease in the PPV to 82%. Specificity was in all steps greater than 99%.

**Figure 2.**
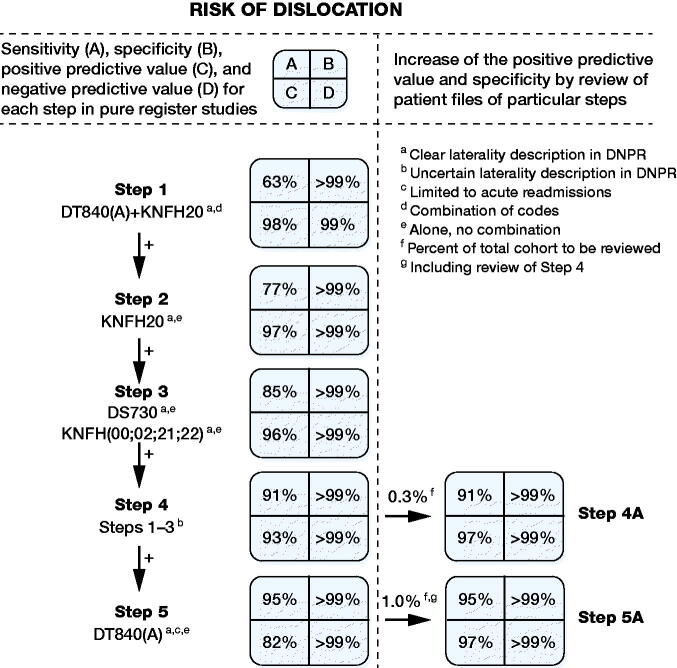
Development of the algorithm for identifying dislocation following primary THA. Flowchart presenting sensitivity, specificity, and predictive values in the attempt to identify the risk of dislocation in a pre-defined cohort of THA patients. Researchers can then decide on what level of, e.g., sensitivity and positive predictive value is acceptable for their specific study design. Flowchart description: For each step, additional codes are added to the previous step, thereby including more codes and increasing sernsitivity at the cost of decreased specificity and positive predictive value. Step 4 and 5 can achieve an increase in positive predictive value if the patient files describing the hospital contact for the specific group are reviewed and the false positives discarded.

Steps 4A and 5A shows an achievable increase in the PPV for these 2 steps if the patient files for the particular step are reviewed. The results from Table 2 were combined into a flowchart (Figure 2), which states the achievable values for pure register purposes and highlights the expected burden of patient file review, which is an option in clinical studies.

## Discussion

We performed a comprehensive nationwide review and validated the applied codes for THA dislocation. We were able to improve the sensitivity by 28% without sacrificing PPV/specificity using our approach by adding alternative and validated codes.

Accurate measurements and truthful monitoring of specific complications are important in order to decrease the risk of complications. Dislocation is a feared complication after hip replacement, leading to pain, anxiety, and reduced quality of life as well as increased costs in the healthcare system. Moreover, re-dislocations happen in 40% to 68% of patients, increasing the risk of reoperation (Brennan et al. 2012, Hermansen et al. 2020). In our study, an acceptable sensitivity of 91%, a specificity of more than 99%, and a PPV of 93% are achievable when combining the most frequently used codes into an algorithm. The algorithm provides information regarding the expected sensitivity, specificity, and predictive values in a stepwise approach.

Importantly, the sensitivity, specificity, and predictive values presented in the algorithm are derived from a specified cohort of primary OA patients. We have not included patients with secondary OA or femoral neck fracture, which is a limitation. However, there is no reason to believe that the results in other such populations would be different than those in the present study.

To our knowledge, codes for hip dislocation have never before been validated in a Danish setting. The accuracy of the DNPR for several other diseases has shown both low to moderate completeness (Nymark et al. 2003, Gundtoft et al. 2015, Jorgensen et al. 2016, Kristensen et al. 2019) and high PPV (Viborg et al. 2017), indicating that great variation is introduced by coding personnel in different specialties. Internationally, there is also significant variation documented between professional hospital coders and orthopedic surgeons for both diagnoses and complications, emphasizing the need for caution when analyzing these data (Mears et al. 2002, Mont et al. 2002). There are unique possibilities for unambiguous linkage and complete follow-up of all patient contacts with the Danish healthcare system. Therefore, our study is based on the application of codes from the broadest range of surgeons possible. Our follow-up was not limited to readmissions to orthopedic departments, as we also reviewed all non-orthopedic readmissions and outpatient emergency room contacts.

Upon reviewing numerous patient files from admission to discharge, it is our experience that the surgeons in most cases are coding correctly. However, the codes labeled in the surgery descriptions are not transferred to the discharge summary on all occasions, which forms the basis of a patient’s DNPR registration. Instead, codes used by the staff who meet the patient in the emergency department are chosen and, often, these codes are assigned by untrained and younger doctors when the diagnosis is not yet verified.

Remarkably, the sensitivity was only 63% when patients with dislocation were identified with a combination of the correct diagnosis, procedure code, and known laterality, thereby missing one-third of the patients (step 1). The majority of the remaining dislocations can be found using either the correct diagnosis or procedure code alone. In particular, the procedure code alone is trustworthy, while the effect of the diagnosis code DT48.0(A) is far more unpredictable. The diagnosis is applied to any kind of mechanical complication, making it widely used in planned outpatient contacts. This supports the inclusion of several false-positive cases if the diagnosis alone is uncritically used in the algorithm without review of every patient case. We included only acute admissions or emergency room contacts with DT840 alone to keep the PPV as high as possible.

With this algorithm, we focused on the risk of dislocation, thus finding all patients with at least 1 dislocation and not necessarily identifying all dislocations for every patient. This is typically useful in larger register settings monitoring complications. In smaller clinical studies with closer follow-up and involvement of patient-reported outcome measures, it may be of greater importance to report all events of dislocation. Our algorithm possesses lower sensitivity for this scenario (step 4: sensitivity = 88% and PPV = 94%; step 5: sensitivity = 95% and PPV = 84%).

In steps 1 to 3 and 5 of the algorithm, laterality is known in both DHR (laterality of the THA) and DNPR (laterality of the hospital contact), which is why a mix in laterality of bilateral THA cases is non-existent. In step 4 we included hospital contacts with unknown or uncertain DNPR laterality to increase sensitivity. A decrease in the PPV is therefore obvious and can be managed by review of a few patient files.

The developed algorithm based on the ICD-10 and NOMESCO codes achieved a sensitivity of 91% and a PPV at 93% using register data alone, which we consider acceptable. As the rates of missed patients with dislocation and false positivity were almost equal, the algorithm gives a precise measure for the risk of dislocation in this study. Higher sensitivity is possible but at the expense of drastically lowering the PPV, which is not feasible for register studies. In perspective, this algorithm is meant for incorporation in national registers for the reliable registration of dislocations and will be of major importance for monitoring this severe complication. Also, because the settings of both hip registers and coding algorithms in other Nordic countries are similar to the Danish, it would be an obvious recommendation also to validate the algorithms within the Nordic Arthroplasty Register Association (NARA) collaboration.

## Supplementary Material

Supplemental Material

## Supplementary data

Appendices A–D ((A) a list of the diagnostic ICD-10 codes, (B) the procedural NCSP codes, which were applied in the National Patient Registry, (C) interpretation of correct coding of a THA dislocation, and (D) description of the review burden related to Table 2) are available in the online version of the article, http://dx.doi.org/10.1080/17453674.2020.1868708
