# Supplementary material for: Development of a diagnostic algorithm identifying cases of dislocation after primary total hip arthroplasty—based on 31,762 patients from the Danish Hip Arthroplasty Register
Source: Acta Orthop. 2021 Jan 13;92(2):137–42. doi: 10.1080/17453674.2020.1868708 (PMC8158188; doi:10.1080/17453674.2020.1868708)
Supplement: Supplemental Material [file IORT_A_1868708_SM3182.pdf]

Supplementary data

Appendix A. Diagnostic ICD-10 codes applied in the National Patient Registry data extraction to identify possible incorrectly coded dislocation cases

DS33.3*	Dislocation of other and unspecified parts of lumbar spine and pelvis
DS70–DS79*	Injuries to the hip and thigh
DT03.1 + DT03.3–DT03.9	Dislocations, sprains and strains involving multiple regions of lower limb(s)
DT09.2	Dislocation, sprain and strain of unspecified joint and ligament of trunk
DT12*–DT13*	Other injuries of lower limb, level unspecified
DT14.3	Dislocation, sprain, and strain of unspecified body region
DT81.9	Unspecified complication of procedure
DT84.0*–DT85*	Complications of internal orthopedic prosthetic devices, implants, and grafts
DT88.9	Complication of surgical and medical care, unspecified
DT93*	Sequelae of injuries of lower limb
DT94*	Sequelae of injuries involving multiple and unspecified body regions
DT98.1–DT98.2	Sequelae of other and unspecified effects of external causes
DM15*–DM16* + DM19*	Polyarthrosis/Arthrosis of hip/Other arthrosis
DM24.3*	Pathological dislocation and subluxation of joint, not elsewhere classified
DM24.4A+B	Recurrent dislocation and subluxation of joint
DM24.9	Joint derangement, unspecified
DM25.2	Flail joint
DM25.3	Other instability of joint
DM25.5	Pain in joint
DM25.9	Joint disorder, unspecified
DM96.6*–DM96.9*	Fracture of bone following insertion of orthopedic implant, joint prosthesis, or bone plate/Other postprocedural musculoskeletal disorders/ Postprocedural musculoskeletal disorder, unspecified
DZ03.9	Observation for suspected disease or condition, unspecified
DZ04.2*–DZ04.3*	Examination and observation following work accident/Examination and observation following other accident
DZ04.9*	Examination and observation for unspecified reason
DZ96.6*–DZ96.7*	Presence of orthopedic joint implants/ Presence of other bone and tendon implants
DZ98.8	Other specified postsurgical states

* = including sublevels

Appendix B. Procedural NCSP codes applied in the National Patient Registry data extraction to identify possible incorrectly coded dislocation cases

KNFA0*–KNFG9*	Exploratory procedures on the hip and thigh/ Primary insertions of joint prosthesis in the hip joint/Secondary insertion of joint prosthesis into the hip joint/Operations on joint capsule and ligaments in the hip joint/Operations on synovia and joint surfaces of the hip joint/Resections, arthroplasty and arthrodesis of the hip joint
KNFJ0*–KNFW9*	Fracture treatments in the femur/Bone operations on the femur/Operations on muscles and tendons in the hip and thigh/Operations on fascia, tendon sheaths, ganglia and bursae in the hip and thighs/Transplantations on hip and thighs/Replantations on hip and thighs/Amputations and other related hip and thigh surgery/ Surgery for hip and thigh tumors/Operations of tendon, joint and bone infections in the hip and thigh/Different hip and thigh surgery/Removal of implants and external fixation equipment from hip and thigh/Reoperations after hip and thigh surgery
KNFH0*–KNFH9*	Different joint operations in the hip
KNE*	Operations on the pelvis
KTNF*	Minor surgical procedures on hip and thighs

Appendix C. Interpretation of correct coding of a THA dislocation

Ideally, every patient contact concerning an radiographically verified THA dislocation should be assigned an ICD-10 diagnostic code (DT84.0A—Mechanical complication of internal joint prosthesis, hip). The contact should likewise be linked with a procedural code describing the intervention being performed, in this case reduction of the dislocated hip. The Danish version of the Nordic Medico-Statistical Committees (NOMESCO) Classification of Surgical Procedures (NCSP) is currently the standard application in Denmark. In this setting, the code KNFH2* (reduction of dislocated joint prosthesis in the hip; *either closed, arthroscopic, or open) would be the most appropriate choice. There is also a laterality variable for each of the two codes. This is particularly important in order to discriminate between relevant hip contacts and a contralateral hip not included in this study.

Appendix D. Description of the review burden related to Table 2

A total of 31,762 THAs are included in our study. Here we have listed the number of contacts in groups 4 and 5, and how many patient files need review to either confirm or invalidate dislocation.

Step 4A: There were 106 contacts in Group 4, and after reviewing we confirmed 70 dislocations and refuted 36.

Step 5A: There were 203 contacts in Group 5, and after reviewing we confirmed 43 dislocations and refuted 160.
